# The refracture of a malunited midshaft clavicle with prominent callus in a 17-year-old: a case report

**DOI:** 10.1097/RC9.0000000000000494

**Published:** 2026-05-11

**Authors:** Sujith Shahul, Bisher Tulimat, Humam Ali, Abdirizak Hussein, Habib Alismaily

**Affiliations:** a Orthopaedics and Trauma Department, Rashid Hospital, Dubai Health, Dubai, United Arab Emirates; b School of Medicine, Royal College of Surgeons in Ireland – Medical University of Bahrain, Busaiteen, Bahrain; c School of Medicine, Ajman University, Ajman, United Arab Emirates

**Keywords:** callus formation, case report, clavicle malunion, internal fixation, orthopedic surgery, refracture

## Abstract

**Introduction and importance:**

Refracture through a malunited clavicle is an uncommon complication, especially in adolescents. However, when it occurs early after conservative management, it brings with it unique technical and clinical challenges.

**Presentation of case:**

A 17-year-old male presented with acute right shoulder pain after falling from a horse. He had previously sustained a displaced right midshaft clavicle fracture 2.5 months earlier, which was managed nonoperatively and had healed in malunion with a prominent callus. Radiographs revealed a refracture through the malunion site. He underwent open reduction and internal fixation using a precontoured distal locking clavicle plate. Intraoperatively, dense callus and sclerotic bone ends complicated the reduction, with the loss of a small butterfly fragment during K-wire manipulation. Postoperative recovery was uncomplicated, with early physiotherapy and stable fixation on follow-up imaging.

**Clinical discussion:**

This case underscores the risk of early mechanical failure through incompletely remodeled bone in adolescents. Prominent hard callus and sclerotic fracture ends may hinder anatomical reduction and fixation. Careful surgical planning is required in such cases.

**Conclusion:**

Significantly displaced midshaft clavicle fractures in adolescents may benefit from early surgical management to reduce the risk of malunion and refracture. Anticipating technical challenges in malunited fractures is essential for optimal outcomes.

## Introduction

Clavicle fractures are common injuries, accounting for approximately 2.6–5% of all fractures and up to 35–45% of shoulder girdle injuries, with the midshaft being the most frequently affected segment^[^1,2^]^. In children and young adults, a conservative approach achieves good results for most midshaft clavicle fractures. However, malunion may arise, resulting in cosmetic deformity, persistent pain, or functional impairment^[^3^]^.

Refracture of a malunited clavicle, particularly within a short interval after initial healing, is rare and presents unique challenges in management. The presence of hard callus and sclerotic fracture ends may complicate surgical reduction and fixation, requiring careful intraoperative planning and technique.

HIGHLIGHTS

- Early refracture through a malunited clavicle occurred within 10 weeks of the injury.
- Dense callus and sclerotic bone ends posed technical challenges during fixation.
- Precontoured locking plate restored length, alignment, and stability.
- Postoperative course was uneventful, with confirmed radiographic union.
- Case supports early surgical fixation in displaced adolescent clavicle fractures.

We present a case of a 17-year-old male who sustained a refracture through a malunited midshaft clavicle only 2.5 months after initial conservative management, resulting in significant surgical challenges due to prominent hard callus formation. To our knowledge, reports describing such early refracture through malunion with intraoperative technical difficulties in adolescents are scarce in the literature. This case was managed in a large government tertiary care hospital. This work has been reported in line with the SCARE 2025 criteria^[^4^]^.

## Presentation of case

On 30 June 2025, a 17-year-old, previously healthy male, presented to the emergency department with acute right shoulder pain following a fall from a horse, during which he sustained direct trauma to his right shoulder.

His medical history was notable for a right midshaft clavicle fracture sustained approximately 2.5 months earlier, which had been managed nonoperatively. That fracture had healed in malunion, resulting in a prominent hard callus and visible deformity. Previous radiographs taken immediately after the initial injury (Supplemental Digital Content Material 1, available at: http://links.lww.com/JS9/H409) demonstrated a displaced midshaft fracture of the right clavicle. Subsequent imaging on 27 May 2025 (Supplemental Digital Content Material 2, available at: http://links.lww.com/JS9/H409) revealed prominent hard callus formation and malunion at the fracture site.

**Figure 1.**
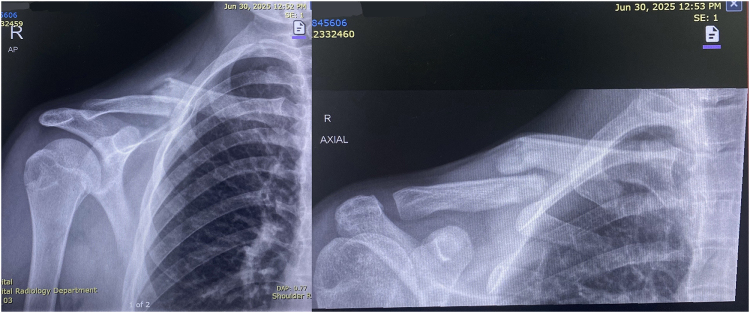
AP view of the right clavicle taken on 30 June 2025, showing a displaced refracture through the site of previous malunion with prominent surrounding hard callus.

On examination, the patient was alert, oriented, and hemodynamically stable. Localized swelling and tenderness were noted over the midshaft of the right clavicle. There was no associated head, neck, thoracic, or abdominal trauma. There was no relevant drug history, family history of bone disease, or psychosocial risk factors such as smoking. The neurovascular examination of the right upper limb was unremarkable. The active range of motion of the right shoulder was significantly restricted due to pain. Additional radiographs obtained on 30 June 2025 (Fig. 1) further confirmed the displaced refracture through the previous malunion site with prominent surrounding hard callus.

**Figure 2.**
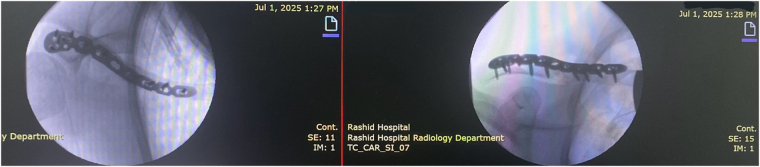
Intraoperative AP and superior views on 30 June 2025 showing reduction and plate fixation of the right clavicle refracture.

Given the displacement and refracture through sclerotic, malunited bone, surgical intervention was planned. The patient underwent open reduction and internal fixation under general anesthesia in the beach-chair position. Intraoperatively, an S-shaped incision was made over the clavicle. A prominent hard callus was encountered, with sclerotic fracture ends and a protruding proximal fragment. The fracture site was debrided and freshened. Preliminary reduction was achieved using a K-wire. Definitive fixation was accomplished using a distal locking clavicle plate (LCP Superior Anterior Clavicle Plate 2.7/3.5 with lateral extension, six holes, right, titanium alloy) secured with both cortical and locking screws. A small butterfly fragment was lost during K-wire removal due to comminution. Fluoroscopic imaging (Fig. 2) confirmed satisfactory alignment and fixation. Hemostasis was achieved, and the wound was closed over a drain. The procedure was performed by an orthopedic specialist with extensive experience in upper limb trauma surgery.

Postoperatively, the patient remained hemodynamically stable. Pain was controlled with multimodal analgesia. Early physiotherapy was initiated, focusing on passive and active-assisted range-of-motion exercises within pain limits. On postoperative assessment, the patient reported right shoulder pain rated 2/10 on a numeric scale. Shoulder motion remained limited (flexion 0–30°, abduction 0–30°, extension 0–10°), with muscle strength measured at 2/5 in flexion, extension, and abduction.

The patient was instructed to wear a right-arm sling for 3 weeks and to maintain a non-weight-bearing status of the right upper limb for 6 weeks.

Follow-up radiographs (Supplemental Digital Content Material 3, available at: http://links.lww.com/JS9/H409) confirmed stable hardware positioning and satisfactory fracture alignment. The patient remained hospitalized for 2 days and was discharged in stable condition. There were no postoperative complications or adverse events during the hospital stay. Long-term follow-up was unavailable, as the patient did not return for further clinic visits.

**Figure 3.**
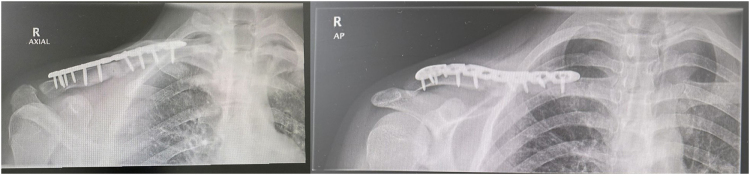
Postoperative right clavicle X-rays (AP and axial views), 31 July 2025, showing united fracture with well-aligned plate and screws.

The most recent radiograph (Fig. 3), performed on 31 July 2025, demonstrated a well-united fracture of the distal mid-shaft of the right clavicle, internally fixed with a plate and screws in good alignment. The right acromioclavicular joint space and margins appeared normal. These findings confirmed successful healing and implant integrity at the 1-month postoperative follow-up.

## Discussion

Midshaft fractures of the clavicle are typically managed conservatively, particularly in adolescents, due to the excellent healing potential of the periosteum and the high rates of bone remodeling in this age group^[^5^]^. However, recent studies have demonstrated that significantly displaced midshaft fractures carry an increased risk of malunion and functional deficits when treated nonoperatively^[^6,7^]^. Malunion may result in shortening of the clavicle, alteration of shoulder mechanics, and scapular dyskinesis, all of which can contribute to chronic pain and reduced upper limb function^[^8,9^]^.

While nonunion of midshaft clavicle fractures is a well-recognized complication, refracture through a malunited site is rarely reported, particularly in adolescents. A study by McKee *et al* noted that residual deformity and biomechanical weakness in malunited clavicles may predispose patients to refracture, especially following direct trauma^[^10^]^. However, most documented refractures occur years after the initial injury, once patients have resumed normal activities, rather than within months, as in our case.

The presence of prominent hard callus and sclerotic bone ends, as encountered in our patient, introduces considerable challenges during surgical fixation. Dense callus may obscure fracture lines and anatomical landmarks, complicating both reduction and hardware placement^[^11^]^. Excessive force during reduction or instrumentation can result in further comminution, as occurred in our patient with the loss of a small butterfly fragment. In such cases, preoperative CT imaging can aid surgical planning by delineating callus morphology and fracture geometry, although this was not performed in our patient^[^12,13^]^.

Biomechanically, malunited clavicles demonstrate reduced load-to-failure strength and may transmit abnormal forces across the shoulder girdle^[^14^]^. Restoration of normal length and alignment through surgical fixation can alleviate symptoms and improve function, particularly in young, active patients^[^8^]^. Contemporary fixation techniques, including precontoured locking plates, provide enhanced stability and may reduce the risk of implant-related complications compared to traditional plating methods^[^15,16^]^.

Our case underscores several important clinical lessons. Firstly, clinicians should maintain a high index of suspicion for refracture in patients with prior clavicular malunion who sustain new shoulder trauma, even relatively soon after initial healing. Secondly, early surgical intervention may be warranted in significantly displaced initial fractures to avoid malunion and subsequent refracture risk, particularly in active adolescents. Finally, surgeons should anticipate intraoperative difficulties associated with dense callus formation and sclerotic bone ends, and plan fixation strategies accordingly.

Although refracture through a malunited callus is rare, this case highlights the potential for early mechanical failure in incompletely remodeled bone and emphasizes the importance of individualized treatment decisions for clavicle fractures in the adolescent population.

## Conclusion

Refracture through a malunited clavicle is an uncommon complication, particularly in adolescents, and may occur earlier than expected following initial healing. Dense callus formation and sclerotic fracture ends can present significant surgical challenges during fixation. This case emphasizes the importance of careful follow-up in patients managed conservatively for displaced clavicle fractures and suggests that early surgical intervention should be considered in cases at risk of malunion to prevent refracture and ensure optimal functional outcomes.

## Data Availability

All data supporting the findings of this study are contained within the manuscript.
